# Non-Coding RNA as Novel Players in the Pathophysiology of Schizophrenia

**DOI:** 10.3390/ncrna4020011

**Published:** 2018-04-12

**Authors:** Andrew Gibbons, Madhara Udawela, Brian Dean

**Affiliations:** 1 The Florey Institute for Neuroscience and Mental Health, 30 Royal Parade, Parkville, VIC 3052, Australia; madhara.udawela@florey.edu.au; 2 The Department of Psychiatry, the University of Melbourne, Parkville, VIC 3010, Australia; 3 The Centre for Mental Health, Swinburne University of Technology, Hawthorn, VIC 3122, Australia; brian.dean@florey.edu.au

**Keywords:** schizophrenia, central nervous system, microRNA, lncRNA, snoRNA, biomarkers

## Abstract

Schizophrenia is associated with diverse changes in the brain’s transcriptome and proteome. Underlying these changes is the complex dysregulation of gene expression and protein production that varies both spatially across brain regions and temporally with the progression of the illness. The growing body of literature showing changes in non-coding RNA in individuals with schizophrenia offers new insights into the mechanisms causing this dysregulation. A large number of studies have reported that the expression of microRNA (miRNA) is altered in the brains of individuals with schizophrenia. This evidence is complemented by findings that single nucleotide polymorphisms (SNPs) in miRNA host gene sequences can confer an increased risk of developing the disorder. Additionally, recent evidence suggests the expression of other non-coding RNAs, such as small nucleolar RNA and long non-coding RNA, may also be affected in schizophrenia. Understanding how these changes in non-coding RNAs contribute to the development and progression of schizophrenia offers potential avenues for the better treatment and diagnosis of the disorder. This review will focus on the evidence supporting the involvement of non-coding RNA in schizophrenia and its therapeutic potential.

## 1. Introduction

Schizophrenia is a debilitating psychiatric disorder diagnosed by the presence of a constellation of symptoms that affects nearly 1% of people over their lifetime. This constellation of symptoms varies between people with schizophrenia, but fits into three categories: positive symptoms (those symptoms not seen in healthy individuals, e.g., hallucinations, delusions, and thought disorder and movement disorder), negative symptoms (reflecting a disruption of normal emotions and behavior, e.g., apathy, alogia, avolition, and affective flattening), and cognitive symptoms (e.g., deficits in executive function and attention) [1]. The aetiology of schizophrenia remains to be fully elucidated, but there is now considerable data to suggest that changes in gene expression make a contribution to the pathophysiology of the disorder. 

Post-mortem studies of the transcriptome and proteome report complex spatio-temporal changes in the expression of a wide array of genes and proteins in the brains of subjects with schizophrenia [2,3,4]. Furthermore, the schizophrenia-related genes highlighted in expression studies are generally not those identified by genetic association studies. Furthermore, microarray studies that have enriched their data analysis for those genes that Genome Wide Association Studies have identified as being associated with schizophrenia report only modest changes in a small number of genes in subjects with schizophrenia [5]. These disparities suggest the biological mechanisms underlying schizophrenia are governed by a complicated dysregulation of gene expression, and there is now a recognition that epigenetic control of gene expression may contribute to this dysregulation [6,7]. Classically, the role of RNA in eukaryotes has been considered to be as an intermediary carrier of the genetic template, facilitating the production of proteins as the sole drivers of cellular function. Thus, RNA was produced by “coding” regions of the genome, whilst “non-coding” regions of the genome were regarded as non-functional genetic debris left over from evolutionary processes [8]. However, it is now understood that non-coding RNA, translated from non-coding regions of the genome, can affect gene transcription and translation, and this has revolutionised our understanding of gene regulatory processes. There is now a growing realisation of the importance of non-coding RNA in the development and maintenance of the central nervous system [9], and their contribution to disorders of the nervous system [10].

Non-coding RNA includes several genetically-distinct families of RNA molecules that have been loosely categorised, based on the size of their mature sequences. Amongst the small (<200 nucleotides) RNAs, there is a growing body of literature showing that microRNAs (miRNA) play a major role in the pathophysiology of schizophrenia [11,12,13]. Recently, however, other families of small RNA, such as small nucleolar RNA (snoRNA), have also been implicated in the disorder [14]. Furthermore, a growing body of evidence suggests long non-coding RNAs (lncRNA) (>200 nucleotides) are also important contributors to schizophrenia [15,16]. This review will focus on the literature that supports a role for non-coding RNA in the pathophysiology of schizophrenia.

## 2. MicroRNA in the Healthy Brain

**Figure 1:**
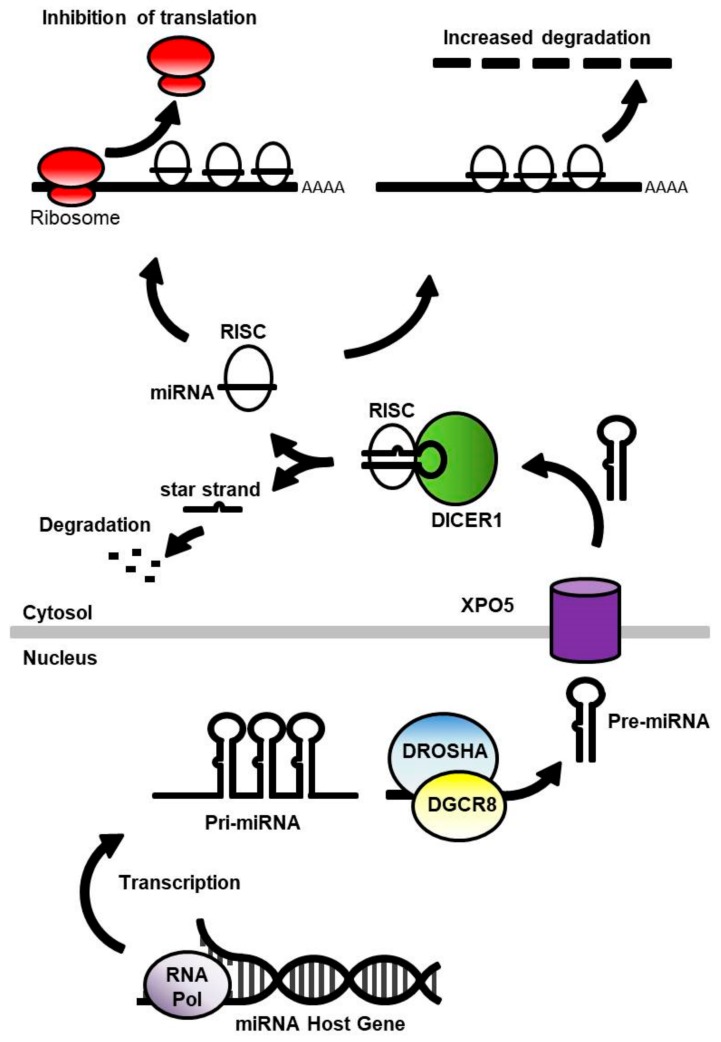
A schematic of microRNA (miRNA) processing and function. miRNA is initially synthesised as a primary (pri-) miRNA gene product containing 1–6 repeat sequences of the precursor (pre-) miRNA, which consists of the mature miRNA sequence connected to the complementary (star) strand by a hairpin loop. The pre-miRNA is cleaved from the pri-miRNA transcript by the DROSHA/DGCR8 enzyme complex and shuttled out of the nucleus by XPO5. The hairpin loop is cleaved from the pre-miRNA by DICER1, and the miRNA is loaded into the RNA-induced silencing complex (RISC), where the star strand is removed, allowing the mature miRNA to bind to the target messenger RNA (mRNA). The miRNA is able to inhibit protein synthesis by obstructing translation by the ribosome or flagging the mRNA for degradation. Abbreviations: DGCR8, DiGeorge syndrome chromosomal region 8; DICER1, endoribonuclease dicer; DROSHA, drosha ribonuclease III; XPO5, exportin-5; RNA Pol, RNA polymerase.

MicroRNAs are a large family of small (20–22 nucleotides) non-coding RNA molecules that play a major role in post-transcriptional regulation of gene expression. They mediate their activity by binding to complementary sequences within the messenger RNA (mRNA) transcript. This, in turn, affects protein translation by modifying the rate of mRNA degradation, to control the time that translatable mRNA remains in the cell, or by physically obstructing the translation the transcript by ribosomes (Figure 1) (Reviewed in [17,18]). The expression and processing of miRNA begins with the transcription of a primary (pri-) miRNA gene product, containing a sequence of one to six precursor (pre-) miRNA repeats. Processing within the nucleus by the enzyme complex of drosha ribonuclease III (DROSHA) and DiGeorge syndrome chromosomal region 8 (DGCR8) releases the pre-miRNA, a double-stranded RNA molecule consisting of the mature miRNA linked to a complementary (star-) strand by a hairpin loop. The pre-miRNA is then exported to the cytoplasm by the nucleocytoplasmic shuttle protein exportin-5 (XPO5). Endoribonuclease dicer (DICER1) then cleaves the hairpin loop, allowing the miRNA to be loaded into the RNA-induced silencing complex (RISC) where the start-strand is removed, and the mature miRNA is able to bind to target mRNA transcripts [19]. 

MicroRNAs are enriched in the human brain compared to other organs, suggesting they are crucial for the proper functioning of the central nervous system (CNS) [20]. A comparison of miRNA expression levels between foetal and adult human post-mortem tissue showed that, amongst the 54 miRNAs measured, total miRNA expression levels in the CNS were nearly three times higher during prenatal development [20]. Similarly, a microarray analysis of miRNA expression in the dorsolateral prefrontal cortex (DLPFC) taken post-mortem from 97 non-psychiatric subjects, ranging in age from neonates to elderly cases, reported that miRNA levels were highest from early infancy and decreased significantly from teenage years onwards [21] following the maturation of neural networks [22]. Furthermore, miRNAs have been shown to target a broad number of genes that are critical for the neurodevelopment and normal brain function [23,24]. Such findings highlight a significant role for miRNAs in regulating the development and maintenance of the nervous system [25,26]. 

## 3. MicroRNAs in Schizophrenia

The growing recognition of the importance of miRNA in regulating gene expression during neurodevelopment has led to an increasing focus on whether the disruption of miRNA regulation could underlie CNS disorders that are characterised by complex changes in CNS-related gene expression, such as schizophrenia [27]. Studies in developing rodents show that miRNAs display complex spatial and temporal patterns of expression [28]. Furthermore, prenatal stress, which has been proposed to confer vulnerability to developing schizophrenia, can affect the expression of miRNAs that are associated with neurodevelopment and brain function [29]. Human post-mortem studies report diverse changes in protein and coding-RNA expression in different parts of the brain in subjects with schizophrenia [30,31,32,33,34]. The distinct patterns of miRNA expression may, in part, explain how such complex changes arise from a common genetic predisposition to the illness. Amongst the first studies to explore miRNA expression in subjects with schizophrenia, Perkins et al. reported lower levels of the miRNA miR-30B in the prefrontal cortex of subjects with schizophrenia [35]. The *MIR130B* gene is located within cytogenetic band 22q11.21. This region is highly implicated in schizophrenia both as the location of several schizophrenia candidate genes, including catechol-*O*-methyltransferase (*COMT*), and due to its involvement in DiGeorge syndrome [36,37,38]. Individuals with DiGeorge syndrome carry deletions spanning chromosomal band 22q11.2 and have an increased risk of developing a psychotic illness, with a quarter of suffers meeting the criteria for schizophrenia under the Diagnostic and Statistical Manual of Mental Disorders [39]. In contrast to the prefrontal cortex, miR-130B expression is reportedly unaltered in the superior parietal lobule in schizophrenia, and no association was found between single nucleotide polymorphisms (SNPs) in the *MIR130B* gene and the incidence of schizophrenia [40]. Interestingly, miR-130B levels are reported to be increase in the blood of patients with schizophrenia, suggesting miR-130B involvement in the disorder involves complex, tissue-specific changes in miRNA expression [41].

Further schizophrenia-associated miRNA from the 22q11.2 region include *MIR185*, a miRNA that was first implicated in schizophrenia through mouse studies. *Df(16)A^+/−^* mice, which are modified to carry a deletion homologous to the DiGeorge syndrome 22q11.2 deletion, show strong downregulation of miR-185 in the hippocampus and prefrontal cortex [42,43]. Furthermore, two miR-185 target genes, *RHOA* and *CDC42* [44], have been implicated in schizophrenia [45,46]. Studies in humans are less convincing, with SNPs in the *MIR185* gene showing no association with schizophrenia [42]. To date, no study has reported whether miR-185 RNA levels are altered in schizophrenia. Patients with chromosome DiGeorge syndrome, however, do show substantial deficits in plasma miR-185 levels, warranting further investigation of this miRNA in patients with schizophrenia [47,48].

Several subsequent studies have examined changes in miRNA expression in the post-mortem CNS from subjects with schizophrenia. These studies have reported changes in various different miRNAs in areas of the frontal [12,13,49] and temporal [50] cortices that neuroimaging studies suggest are affected in schizophrenia [51,52,53]. Microarray analyses of miRNA levels in the superior temporal gyrus and DLPFC report a general pattern towards increased miRNA expression in the CNS of subjects with schizophrenia. These studies show that the expression of 21% of the miRNAs expressed in the superior temporal gyrus (STG) and 9% of the miRNAs expressed in the DLPFC is increased in subjects with schizophrenia compared to controls [11]. This suggests that schizophrenia involves the disruption of a substantial proportion of the brain’s miRNA-mediated transcriptional regulation. Notably, in silico pathway analysis and in vitro studies suggest that the miRNAs that are altered in schizophrenia are able to regulate the expression of several schizophrenia candidate genes involved in important neurotransmitter signalling and neurodevelopmental pathways [11,54,55]. This may explain the dysregulation of key molecular components across a broad array of neural signalling pathways that are reported to occur in schizophrenia.

## 4. miR-137 in Schizophrenia

Studies into the miRNA miR-137 have provided the strongest evidence for the involvement of a single miRNA in schizophrenia. *MIR137* is located on chromosome 1p22 located within the host gene sequence *MIR137HG* and the overlapping noncoding genes *AK3011400* and *AK309618*. A number of studies have reported an association between the SNPs in the *MIR137* gene and the incidence of schizophrenia, with the T allele at the *MIR137* SNP rs1625579 being most consistently associated with an increased risk of schizophrenia [56,57,58,59]. By contrast, some other studies have failed to show an association of any *MIR137* SNPs and schizophrenia [60,61,62]. However, a meta-analysis of 12 case-control studies showed an association between rs1625579 and the incidence of schizophrenia, reporting that the T allele is associated with a 15% increase in the risk of developing schizophrenia compared to the G allele and that patients with the T/T genotype were at a 32% greater risk developing schizophrenia compared to those with the G/G genotype [63]. This finding has been supported by another meta-analysis that showed an association between the T allele and the homozygous T/T genotype and the incidence of schizophrenia, but suggested that T allele carriers were at no greater risk of developing schizophrenia than the subjects with the G/G genotype [64]. Post-mortem studies report that the expression of miR-137 is not altered in the DLPFC of subjects with schizophrenia compared to non-psychiatric controls. When expression is examined in relation to rs1625579 genotype, control subjects who are homozygous for the T allele have lower levels of miR-137 RNA compared to G/T and G/G genotypes. miR-137 RNA levels in T/T genotype subjects with schizophrenia, however, are not significantly different from those in G/T and G/G genotype cases [65]. 

Deficits in miR-137 appear to be associated with changes in connectivity rather than large structural effects in the brain. Progressive cortical loss of grey matter and a disruption of white matter tracts has been reported in patients with schizophrenia [66,67]; however, neuroimaging studies do not show an association between SNPs in the *MIR137* gene and the loss of grey matter or white matter volume across the brain [68,69]. Interestingly, one study has reported that rs1625579 T/T genotype subjects with schizophrenia have a smaller reduction in corpus callosum volume compared to G/G and G/T genotype patients [70]. Furthermore, another study has reported an association between the T/T genotype and a reduction in occipital, parietal, and temporal lobe grey matter volume in patients with schizophrenia, although the strength of this relationship is dependent upon the cumulative presence of risk alleles for the miR137 regulated genes transcription factor 4 (*TCF4*), prostaglandin-endoperoxide synthase 2 (*PTGS2*), mitogen-activated protein kinase 1 (*MAPK1*)*,* and mitogen-activated protein kinase 3 (*MAPK3*) [71]. This suggests the penetrance of *MIR137* as a candidate gene for schizophrenia is likely to be dependent on the interaction with other genes.

The targeted overexpression of miR-137 in the mouse hippocampus shows that miR-137 can alter synaptic vesicle release, affecting long term potentiation and synaptic plasticity. These mice also show impairments in hippocampal-based learning [72]. A neuroimaging study reported that working memory performance and response times of patients with schizophrenia, assessed using the Steinberg item recognition paradigm (SIRP), did not significantly vary with rs1625579 genotype. However, patients with the T/T genotype showed hyper-activation on the DLPFC compared to patients with the G/G or G/T genotype, suggesting that neural processing may be less efficient in rs1625579 T allele homozygotes [73]. This breakdown in the efficiency of neural connectivity with rs1625579 genotype is supported by a study in healthy subjects that showed that subjects with the T/T rs1625579 genotype do not show the normal correlation between working memory performance and DLPFC-hippocampal connectivity that is seen in subjects with the G/T genotype. This suggests that miR-137 is important in establishing connections between the DLPFC and the hippocampus [74]. Conversely, the rs1625579 T/T genotype is associated with increased connectivity between the frontal cortex and amygdala, suggesting miR-137 may have much wider effects on brain connectivity [75].

Functionally, most reports suggest the rs1625579 genotype impacts cognitive function. In patients with schizophrenia, the T/T genotype is associated with working memory deficits, with patients showing poor performance on the brief assessment of cognition in schizophrenia (BACS) [58,59]. These subjects also show worse negative symptoms on the positive and negative syndrome scale (PANSS) [58]. Even amongst studies that fail to find an association between *MIR137* SNPs and the incidence of schizophrenia, some studies have shown a correlation between rs1625579 genotype and cognitive performance. In a study of combined schizophrenia and schizoaffective disorder patients, while rs1625579 genotype was not found to be different between psychiatric cases with cognitive deficits, the T/T genotype could predict cognitive deficits in patients with increasing negative symptoms [76]. Another study of patients with schizophrenia, bipolar disorder, and schizoaffective disorder also reported no association between rs1625579 genotype and the incidence of psychiatric illness, but the ‘T’ risk allele was associated with lower cognitive performance. Interestingly, the T allele was also associated with a lower operational criteria checklist for psychotic illness (OPCRIT) scores and a lower lifetime measure of psychosis-symptom incongruity, suggesting that this allele is associated with fewer psychotic symptoms [77]. 

## 5. Disruption of MicroRNA Processing Machinery

The changes in expression across a diverse range of miRNAs in schizophrenia, reported by microarray studies in the post-mortem brain [11], are suggestive of a broader disruption of the miRNA processing machinery. The location of DiGeorge critical region gene 8 (DGCR8), part of the enzyme complex that cleaves the pri-miRNA gene product into pre-miRNA, within the chromosome 22q11.2 schizophrenia-susceptibility region has given considerable weight to this idea. Rodent studies report that *Dgcr8*^+/-^ mice, which are heterozygous for an ablated copy of the *Dgcr8* gene, have impairments in synaptic plasticity and synaptic potentiation in the prefrontal cortex compared to wildtype mice [78]. These mice also display deficits in spatial working memory and reduced pre-pulse inhibition, an endophenotype of schizophrenia that is seen in both rodents and humans, and which is used to model the disorder [43].

Genetic association studies have reported associations between SNPs in *DGCR8* (rs3757, rs8139591, rs9606248) and the pre-miRNA processing gene *DICER1* (rs3742330, rs11621737) and the incidence of schizophrenia in Han Chinese patients [79,80]. However, while another study in a Han Chinese cohort found marginal associations between some miRNA processing genes and the incidence of schizophrenia, different association testing methods were shown to produce inconsistent results, and there was no strong evidence of any variants in any genes involved in miRNA processing being associated with schizophrenia [81].

In post-mortem studies, dysregulation of miRNA processing-gene expression in schizophrenia varies between brain regions. In Brodmann area (BA) 9 of the DLPFC, DGCR8, DROSHA, and DICER1 have all be reported to be increased in schizophrenia with other miRNA processing proteins, such as XPO5, which is reported as unchanged [11]. By contrast, in the adjacent BA 46 of the DLPFC, only DICER1 mRNA is shown to be significantly upregulated in in schizophrenia [13]. Conversely, only DGCR8 mRNA is significantly increased in the STG of subjects with the disorder [11]. Such regional differences in the genes processing miRNA could produce different rates of miRNA processing in different parts of the brain in people with schizophrenia. This could contribute to regional specificity of altered protein expression often reported in schizophrenia, particularly where such changes are not easily attributed to altered gene expression [82]. 

## 6. Short Non-Coding RNA in Schizophrenia

Beyond miRNA, some studies have recently suggested other small RNAs may be affected in schizophrenia. Deep sequencing of synaptosomes purified from the frontal pole revealed a 50% decrease in the level of the snoRNA SNORD85 in subjects with schizophrenia compared to controls [83]. Deficits in the level of Y3, a Y RNA involved in DNA replication processing of histone transcripts [84,85], were also seen in synaptosomes in schizophrenia. However, these small non-coding RNAs were identified from a small number of subjects and require further validation [83]. A recent promoter methylation microarray conducted on blood genomic DNA from two sets of female twins who were discordant for schizophrenia reported increased methylation in the *SNORD115* and *SNORD116* snoRNA gene clusters [86]. A subsequent RNA sequencing (RNA-Seq) expression analysis of small RNAs in the post-mortem anterior cingulate from subjects with schizophrenia showed differential expression of probes within SNORD115 and SNORD116 between female schizophrenia and control subjects, but not across the diagnosis as a whole, suggesting snoRNA involvement in schizophrenia is sex specific [14]. The snoRNA family of small non-coding RNA is comprised of short RNA sequences that typically drive the 2’-O-methylation and pseudouridylation of non-coding RNAs, such as ribosomal RNA and transfer RNA. However, contrasting most snoRNAs, SNORD115 and SNORD116 do not bind to classical snoRNA-associated proteins but instead appear to regulate the alternative splicing of gene transcripts [87,88]. 

To date, the way SNORD115 and SNORD116 mediate their effect in schizophrenia is not clear. It is significant that the RNA-Seq study that showed changes in SNORD115 and SNORD116 expression in females with schizophrenia also showed changes in the levels of the snoRNA U2, a component of the spliceosome that removes intronic sequences from precursor-mRNA in females with schizophrenia [14]. This would support a role for these snoRNAs in the dysregulation of alternative splicing, which is thought to be affected in schizophrenia [89,90]. SNORD115 shares sequence complementarity with ExonVb of the serotonin 2C receptor (HTR2C) transcript, and is thought to modulate alternative splicing of HTR2C [87]. While decreased HTR2C levels and function have been reported in subjects with schizophrenia [91,92], there does not appear to be any evidence of altered RNA editing of HTR2C in the CNS of subjects with schizophrenia [93,94]. Rodent studies suggest both SNORD115 and SNORD116 appear to be involved in memory consolidation during fear-based contextual learning, and thus they may contribute to the cognitive deficits in schizophrenia [95,96].

## 7. Long Non-Coding RNAs in Schizophrenia

**Figure 2:**
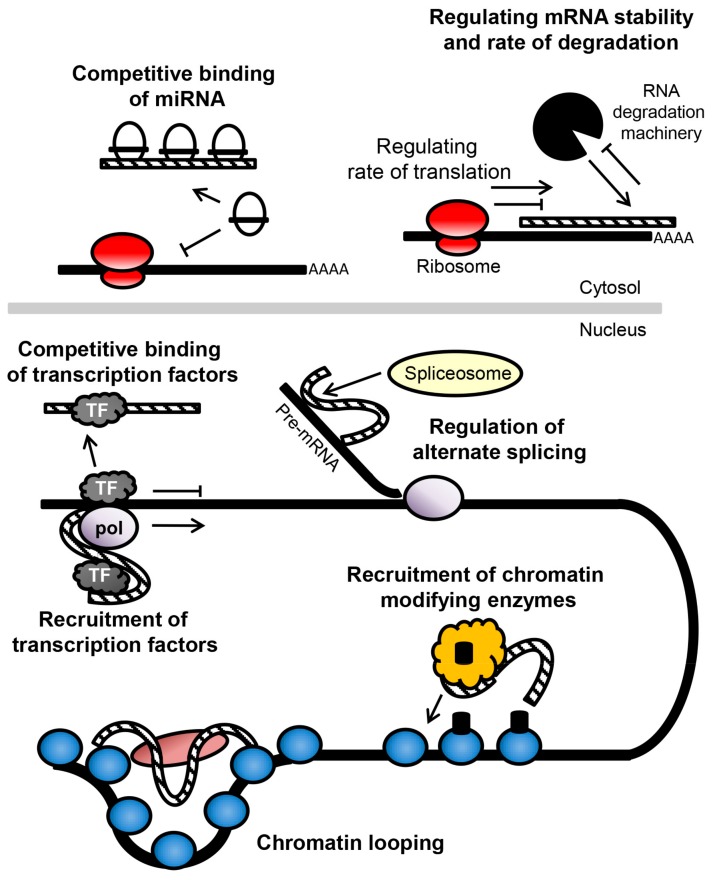
A summary of the mechanisms of action of long non-coding RNA (lncRNA). Long non-coding RNA (dashed line) can act to regulate translation of mRNA by (i) controlling miRNA availability by competing with miRNA target sites on the mRNA or (ii) binding to the mRNA to control the rate of translation or RNA degradation. Within the nucleus, lncRNA can affect transcription by (iii) acting as decoy to inhibit transcription factor availability or (iv) recruiting the transcription factors to the promoter site, or by (v) regulating alternative splicing of the transcript. lncRNA can also affect epigenetic state and chromosome structure by (vi) recruiting chromatin modifying enzymes to the DNA and (vii) controlling chromatin looping. Abbreviations: TF, transcription factor; pol, RNA polymerase.

Long non-coding RNAs are a large and diverse group of non-coding RNA over 200 nucleotides in length. Long non-coding RNA represents the majority of the non-coding transcriptome and use several mechanisms to mediate their activity. Amongst their modes of action, lncRNAs are involved in regulating transcription through the inhibition or recruitment of transcription factors [97,98], by controlling alternate splicing of the mRNA transcript [99], by interacting with chromatin to affect the DNA structure and epigenetic state [100,101], and by affecting the translation and stability of mRNA via binding to complementary transcripts or removing miRNA [99,102,103] (Figure 2).

Several transcriptome-wide array studies have reported altered lncRNA expression profiles in both the periphery and the CNS of subjects with schizophrenia [15,16,104,105]. Amongst these lncRNAs there is strong evidence to support the involvement of the lncRNA myocardial infarction associated transcript (MIAT) in schizophrenia. The *MIAT* gene is located on chromosome 22q12.1, in close proximity to the chromosome 22q11.2 schizophrenia candidate region. Initial investigations into its role in schizophrenia reported lower levels of the MIAT transcript in the STG in subjects with schizophrenia compared to controls [106]. Subsequent studies report that the G to T polymorphism at the *MIAT* SNP rs18944720 is associated with an increased risk of paranoid schizophrenia [107]. Within the CNS, MIAT is reported to be expressed in neuronal populations in which the mature transcript is localised to the nucleus [108,109] and mediates its activity by binding to the splicing factors, SF1, QKI, SRSF1, and CELF, supporting a role for MIAT in regulating alternative splicing in schizophrenia [106,110,111]. Notably, in vitro knockdown of the MIAT transcript in neuronal stem cells is reported to increase the levels of the disrupted in schizophrenia 1 (DISC1) splice variant transcripts DISC1 Esv1, DISC1 Δ3, and DISC1 Δ7Δ8, as well as the neuregulin 1 receptor (ERRB4) transcript variants ERBB4 CYT-1 and ERBB4 JM-a, but does not alter the expression of the full length isoforms of either gene [106]. These expression patterns of the DISC1 and ERRB4 splice variants are comparable to the increased DISC1 and ERRB4 splice variant levels reported in the post-mortem hippocampus from subjects with schizophrenia [112,113]. Thus, the reported loss of MIAT transcript in schizophrenia may regulate the changes in DISC1 and ERBB4 splice variation seen in subjects with schizophrenia. 

The screening of other regions of the genome associated with the risk of schizophrenia has led to the discovery of other lncRNAs that may be involved in schizophrenia. Genome-wide association studies (GWAS) have reported that polymorphisms in chromosome 1p21.3 are associated with schizophrenia. This association with schizophrenia has been attributed to the miRNA miR-137. However, recent bioinformatic analysis of the chromosome 1p21.3 identified a novel, CNS-expressed lncRNA, EU358092, that may be also be involved in schizophrenia [114]. While the function of this lncRNA has yet to be determined, several schizophrenia GWAS SNPs were reported within the *EU358092* gene sequence, including two SNPS within the predicted regulatory elements of the gene. EU358092 was also reported to show altered expression in SH-SY5Y human neuronal cells in response to psychoactive drugs [114], suggesting the potential for EU358092 to affect molecular pathways relevant to schizophrenia.

## 8. Non-Coding RNAs as Therapeutic Targets

In light of the recentness of studies examining the role non-coding RNA plays in schizophrenia, the therapeutic potential of targeting non-coding RNA to treat this disorder remains largely unexplored. While anti-miRNA and RNA interference agents are currently in phase I trials for the treatment of cancers [115], the difficulty of developing compounds that efficiently cross the blood brain barrier presents a major obstacle towards antipsychotic drugs that can directly target non-coding RNA in the brain. A recent study used liposome nanocapsules to deliver silencing RNA across the blood brain barrier in mice, offering the potential for targeted delivery of RNA transcripts that target or mimic non-coding RNA to the CNS [116]. The growing interest in the therapeutic use of exosomes, as an innate mechanism of intercellular RNA transport, may provide new opportunities for the effective delivery of non-coding RNA-based therapeutic agents to the CNS [117]. By conjugating exosomes with the integrin inhibitor, Cyclo(RGDyK), Tian et al. (2018) were able to target exosomal drug delivery to integrin *α*V*β*3-expressing cells in a mouse model of cerebral ischemia [118]. Such findings suggest exosome-based drug delivery may provide avenues to not only deliver therapeutic non-coding RNA across the blood brain barrier but also target discrete cell populations within the CNS by tagging the exosomes with ligands to cell-specific, surface antigens. To date, there is no published literature supporting developments in this field for treating schizophrenia; however, there is evidence to suggest current antipsychotic drugs can modulate the expression of non-coding RNA. Perkins et al. reported that the expression of the miRNA miR-199a, miR-128a, and miR-128B was increased in the anterior medial frontal cortex of rats following treatment with the typical antipsychotic haloperidol [35]. Although this study showed these miRNAs were not altered in the CNS of subjects with schizophrenia, it provided the first evidence that traditional antipsychotic drugs could alter levels of miRNA in the brain.

Amongst the miRNA shown to be altered in schizophrenia, treating mice with haloperidol and the atypical antipsychotic olanzapine have been shown to decrease expression levels of miR-339, miR193, miR-223, and miR-544 [119]. The reported increase in expression of miR-339 in the STG and miR-193, miR-223, and miR-544 in the DLPFC of subjects with schizophrenia suggests antipsychotics could help restore disrupted miRNA levels in the CNS [11,13,55]. By contrast, some reported changes in non-coding RNA levels in response to antipsychotics do not easily correspond to a therapeutic effect. miR-22 is reportedly lower in the DLPFC of subjects with schizophrenia [12]. However, treatment with haloperidol and the atypical antipsychotic olanzapine has been reported to also reduce miR-22 expression in both mice and in cultured neuronal cells [119,120]. Furthermore, it is unclear whether modulating these miRNAs relates to the therapeutic effects of the drugs. 

Some evidence that modulating non-coding RNA may have some therapeutic benefit was reported by a quantitative polymerase chain reaction (qPCR) study examining plasma levels of miRNA in unmedicated patients with schizophrenia. Levels of the miRNAs miR-181B, miR-30E, miR-34A, and miR-7 were found to be increased in patients with schizophrenia. Of these miRNAs, miR-181B levels were reduced after 6 weeks of treatment with antipsychotics. Furthermore, miR-181B levels were correlated with improvements in negative symptoms in PANSS performance [121]. Similarly, two microarray studies examining miRNA and lncRNA in peripheral blood mononuclear cells from unmedicated patients with schizophrenia showed that levels of the miRNA miR-21 and lncRNAs NONHSAT041499 and NONHSAT089447 were increased in in patients compared to healthy controls, and antipsychotic treatment significantly reduces the levels of all three non-coding RNAs. The decrease in miR-25 and NONHSAT041499 levels was also significantly correlated with improved PANSS performance, although in this case, most notably with improved positive symptoms with miR-25 also correlating with improvements in general psychopathology [15,122]. While it is unknown whether these changes correspond to changes in these non-coding RNAs in the CNS, these studies do suggest that modulating non-coding RNA may be associated with improved symptom outcome for people with schizophrenia.

## 9. Non-Coding RNAs as Peripheral Biomarkers of Schizophrenia

Delays in effective treatment of psychosis and patient non-compliance have been shown to significantly impact the prognosis of schizophrenia [123,124]. As such, a major goal to psychiatric research is to identify clinical biomarkers that will allow early diagnosis of schizophrenia and predict the treatment response and the risk of adverse effects to ensure the best patient outcome [125]. As in the CNS, individuals with schizophrenia display differential expression of non-coding RNA in the periphery compared with non-psychiatric subjects [126,127]. While the changes in RNA levels in the periphery do not necessarily reflect corresponding changes in expression in the CNS, peripheral non-coding RNA is currently being investigated as a potential biomarker of schizophrenia. Several miRNA have been examined as possible biomarker candidates; however, most studies are conflicting in the sets of miRNA they report as potential biomarkers [41,128,129]. Amongst a panel of miRNA implicated in schizophrenia by post-mortem and clinical studies, Camfurt et al. reported that the levels of miR9, miR-29, miR-106B, miR-125A, and miR-125B were increased in plasma from patients with schizophrenia [130]. Notably, the levels of miR-106B and miR-125, which target vesicular glutamate transporter 1 and glutamate receptor interacting protein 2 respectively, are increased in the post-mortem cortex, suggesting a possible relationship between these miRNA in the plasma and abnormal glutamate regulation in the CNS of people with schizophrenia [11,35]. By contrast, Sun et al. reported that miR-181B, miR-30E, miR-34A, miR-346, and miR-7 levels were increased in plasma from patients with schizophrenia; however, they showed that greater diagnostic specificity could be achieved by assessing these miRNA as a combined panel of biomarkers, rather than individually [131].

Non-adherence to antipsychotic medication is a significant risk factor for relapse of psychotic episodes and is reported to be as high as 40–50% in people with schizophrenia [132]. The severity of adverse side effects of antipsychotic drugs is a significant contributor of non-adherence. Therefore, identifying biomarkers that predict a patient’s adverse response to antipsychotics could be useful indicators of potential non-adherence [133]. An analysis of SNPs within the miRNA target sites of several schizophrenia candidate genes in patients with the disorder found that SNPs in the genes *SNC1A* (rs10497275/miR-1286), *GLCM* (rs17881908/miR-582), *MTHFR* (rs4846049/miR-555), *PIP4K2A* (rs10734041/miR-602), and *CLDN5* (rs756654/miR-486) were associated with the risk of developing tardive dyskinesia, a movement disorder that develops as a side-effect of prolonged antipsychotic use [134]. Such associations could be useful for identifying patients at risk of adverse response to drugs. This will allow for more appropriate drug choice or closer monitoring during treatment to avoid the side-effects of the medication and the risk of non-compliance due to those side-effects. 

Approximately 30% of people with schizophrenia fail to respond to conventional dopaminergic antipsychotics medication [135]. The identification of treatment resistance in schizophrenia is a key objective of biomarker research, to reduce delays in prescribing more effective medication, such as clozapine. As with attempts to identify biomarkers for diagnosing schizophrenia, attempts to identify non-coding RNA biomarkers of treatment resistance have proposed both common and differing sets of RNA biomarkers. Alacam et al. showed that plasma levels of miR-181B, miR-195, and miR-301A are increased in treatment-resistant patients and decreased in treatment-responsive patients compared to controls [136]. Another study also identified plasma levels of miR-181B as a potential biomarker of symptom improvements and treatment response, but suggested this mRNA can most reliably predict treatment resistance, as part of a combined biomarker panel of miRNA that included miR-30e, miR-132, and miR-432 [131]. Interestingly, miR-30e, which has been proposed as a biomarker of both schizophrenia and treatment resistance, and which has undergone phase II clinical trials as a biomarker, was shown to be more sensitive to differentiating between patients with schizophrenia and controls when measured in plasma than in peripheral blood mononuclear cells [137]. This suggests that schizophrenia-related changes in non-coding RNA levels are not uniform throughout the periphery. Additionally, a study in rats showed that the miRNA profile of plasma differed between plasma derived from venous blood vs. arterial blood, providing a potential explanation for the differences in miRNA biomarkers identified by different studies [138]. Therefore, if non-coding RNA is to be used as an effective biomarker for schizophrenia, there will need to be careful consideration of how blood collection protocols are standardised. 

## 10. Concluding Remarks

The noncoding transcriptome imparts an extra layer of complexity to the traditional dogma that proteins alone control the synthesis of proteins from the genetic code. In the decade since the first reports of miRNA abnormalities in schizophrenia [35,40,139], a large body of literature has been published that points to a major disruption of non-coding transcriptome RNA in the CNS and periphery of people with the disorder. These studies should not be viewed merely in addition to existing reports of a disrupted genome, coding-transcriptome, and proteome in schizophrenia [140] but may also provide context as to how changes at the level of gene, mRNA, and protein are related to each other. It is notable, however, that many studies reporting differential expression of non-coding RNA in schizophrenia in the CNS and periphery often identify different sets of non-coding RNA as being most affected in the disorder (Table 1). Post-mortem studies that have examined non-coding RNA expression in different CNS regions from the same subjects show that differential non-coding RNA expression profiles in schizophrenia vary with brain region [11]. Thus, such differences can in part be explained by differences in the CNS regions studied or the type of peripheral samples collected for analysis. However, such discrepancies can be seen even when similar tissue samples are examined. Thus, differences in other demographic factors such as the average age, sex, and ethnic profile of the cohorts may contribute to the genetic and epigenetic heterogeneity of non-coding RNA expression in schizophrenia, and such factors need to be considered when comparing studies.

Studies supporting the potential use of non-coding RNA as biomarkers, not only for psychiatric diagnosis but also for predicting a patient’s response to antipsychotics [131], suggest non-coding RNA may be valuable for understanding important molecular differences between sub-groups of people with the disorder. Further research into the role non-coding RNA plays in schizophrenia may provide insights into the molecular underpinnings of sex differences in schizophrenia, as well sub-types of schizophrenia within the disorder. Sex differences in the incidence, symptom profile, and progression of schizophrenia are widely recognised, and there is now considerable interest into the role that sex hormones, such as estrogen, play in schizophrenia [141]. Several non-coding RNA from different small RNA families show sex-specific, differential expression in the cortex of subjects with schizophrenia [14]. Significantly, the level of the miRNA miR-30B, which can be upregulated in response to estrogen, is increased in the DLPFC of female but not male subjects with schizophrenia [142]. Additionally, miR-30B levels in these subjects are correlated with the genotype of the rs2234693 SNP in the estrogen receptor-*α* gene (*ESR1*). Furthermore, females with schizophrenia have less severe deficits in cognitive parameters, such as memory, compared to males [143], and both miR-30B and ESR1 have been shown to be important for cognitive function [144,145,146,147]. Thus, miR-30B warrants further study to understand whether it may be protective against cognitive deficits seen in schizophrenia.

Beyond sex differences, differential expression of non-coding RNA in schizophrenia has in one instance been shown to differentiate between molecular subpopulations with the illness. Schizophrenia has been proposed to be a syndrome of molecularly distinct disorders [148]. Deficits in cortical muscarinic M1 receptor (CHRM1) protein have been identified in a sub-group of over a quarter of individuals with schizophrenia [149,150]. However, levels of CHRM1 mRNA were not different between schizophrenia cases with CHRM1 deficits compared to those with normal levels of CHRM1 protein [151]. Post-mortem studies have reported that the expression of miR-107, a miRNA that targets the CHRM1 sequence, is increased in schizophrenia [13]. Furthermore, miR-107 levels are shown to only be higher in subjects with deficits in CHRM1 protein, suggesting these deficits might be modulated by miRNA rather than gene expression [152]. The levels of miR-107 do not appear to be altered in the periphery of patients with schizophrenia, limiting its potential as a biomarker [130]. However, it does point to a novel mechanism of CHRM1 regulation that may be useful for further characterising the factors contributing to this sub-type of schizophrenia.

A future challenge will be in understanding how changes in the non-coding RNA of the periphery can offer insights about changes in the CNS. Care must be taken when predicting biological implications of altered miRNA in the brain from observations in the periphery. Estimates of the proportion of known RNA transcripts shared between the blood and brain range from 35% to 80%, with level of RNA in the CNS compared to the periphery being weakly to moderately correlated [152]. Some studies that have compared miRNA in the periphery and the CNS found that miRNA that show altered expression in the periphery of patients with schizophrenia are not altered with diagnosis in the post-mortem CNS [153,154]. However, in a prodromal study of subjects with a high risk of psychotic illness, changes in the miRNA profile of peripheral leucocytes correlated to the rate of cortical thinning subjects who converted to psychosis [155]. This would suggest that, while there may be differences between the non-coding RNA that are altered in the periphery vs. the CNS, changes in non-coding RNA in the periphery do appear to be associated with schizophrenia-related changes in the brain. Furthermore, as miRNA has been shown to be differentially expressed in cortical exosomes in post-mortem subjects with schizophrenia [49], and such exosomes are potentially secreted into the cerebrospinal fluid (CSF) and blood [156,157], the non-coding RNA biomarkers from brain-derived exosomes in the periphery may provide valuable insights into the non-coding RNA profile in the brain comparable to the “liquid biopsies” employed in cancer diagnostics [158]. Thus, future research will need to focus on the biological and functional implications of the altered non-coding RNA expression profiles in schizophrenia and understand the factors regulating non-coding RNA, both in the CNS and peripheral biomarkers.

## Figures and Tables

**Table 1:** A summary of microRNA (miRNA) expression studies showing the range of different miRNA reported as differentially expressed in schizophrenia. MicroRNA in bold are reported to be altered in more than one study.

Study	Tissue Source	Altered RNA Expression in Schizophrenia vs. Control
Alacam et al., 2016 [136]	Plasma	Increased in treatment-resistant schizophrenia: **miR-181b, miR-195**, miR-301 Decreased in treatment-responsive schizophrenia: **miR-181b, miR-195**, miR-301
Banigan et al., 2013 [49]	Prefrontal cortex exosomes	Increased: miR-497 Not changed: miR-15b, miR-29c, miR-31, miR-149, miR-219
Beveridge et al., 2008 [50]	Superior temporal gyrus	Increased: **miR-181b** Not changed: Let7g
Beveridge et al., 2010 [11]	Prefrontal cortex	Increased: Let7d, miR-128, miR-16, miR-181a, **miR-181b**, miR-20a, miR-219, miR-27a, miR-29c, **miR-7**
	Superior temporal gyrus	Increased: **miR-107**; miR-15a, miR-15b, **miR-195**, **miR-181b**, Let7e, miR-20a, **miR-26b** Not changed: miR-16, miR-19a
Burmistrova et al., 2007 [40]	Superior parietal lobule	Not altered: miR-130b
Guella et al., 2013 [65]	Prefrontal cortex	Not changed: miR-137; decreased in rs1625579-T/T control but not schizophrenia cases compared to G/T & G/G genotype
Kim et al., 2010 [55]	Prefrontal cortex	Increased: **miR-34a, miR-132, miR-212**, miR-544, **miR-7**, miR-154
Lai et al., 2016 [153]	Peripheral blood mononuclear cells	Increased: **miR-34a**, miR-449a, miR-564, miR-548d
	Prefrontal cortex	Not changed: miR-34a
	Stiatum	Not changed: miR-34a
Melios et al., 2012 [142]	Prefrontal cortex	Decreased: **miR-30b** in females only
Perkins et al., 2007 [35]	Prefrontal cortex	Decreased: **miR-30b, miR-26b**, miR-92, miR-24, **miR-30e** Not changed: miR-29b, miR-195, miR-7
Santarelli et al., 2011 [13]	Prefrontal cortex	Increased: mi-R17, **miR-107, miR-134**, miR-328, miR-382, miR-652 Not changed: miR-150, miR-199a, miR-25, miR-487a
Shi et al., 2012 [126]	Serum	Increased: **miR-181b**, miR-219, miR-1308, Let7g, **miR-346**, Decreased: **miR-195** Not changed: miR-103
Sun et al., 2015 [131]	Plasma	Increased: **miR-30e, miR-181b, miR-34a, miR-346, miR-7**
Sun et al., 2015 [137]	Plasma	Increased: **miR-132, miR-195, miR-30e, miR-7**
	Peripheral blood mononuclear cells	Increased: **miR-212, miR-34a, miR-30e**
Wei et al., 2015 [41]	Plasma	Increased: miR-130b, miR-193a Not changed: miR-122, miR-130a, miR-13b, miR-193a, miR-502, miR-652, miR-886
Yu et al., 2015 [128]	Peripheral blood mononuclear cells	Increased: **miR-132, miR-134**, miR-1271, miR-664, miR-200c, miR-432
